# M-protein based vaccine induces immunogenicity and protection from *Streptococcus pyogenes* when delivered on a high-density microarray patch (HD-MAP)

**DOI:** 10.1038/s41541-020-00222-2

**Published:** 2020-08-07

**Authors:** Jamie-Lee S. Mills, Cesar M. Jayashi Flores, Simone Reynolds, Christine Wun, Ainslie Calcutt, S. Ben Baker, Senthil Murugappan, Alexandra C. I. Depelsenaire, Jessica Dooley, Paul V. Fahey, Angus H. Forster, Manisha Pandey, Michael F. Good

**Affiliations:** 1 Institute for Glycomics, Griffith University, Gold Coast, Australia; 2 Vaxxas Pty Ltd, Translational Research Institute, Woolloongabba, Australia

**Keywords:** Biotechnology, Microbiology, Medical research, Adaptive immunity, Infection

## Abstract

We evaluated vaccination against *Streptococcus pyogenes* with the candidate vaccine, J8-DT, delivered by a high-density microarray patch (HD-MAP). We showed that vaccination with J8-DT eluted from a coated HD-MAP (J8-DT/HD-MAP), induced similar total IgG responses to that generated by vaccination with J8-DT adjuvanted with Alum (J8-DT/Alum). We evaluated the effect of dose reduction and the number of vaccinations on the antibody response profile of vaccinated mice. A reduction in the number of vaccinations (from three to two) with J8-DT/HD-MAP induced comparable antibody responses to three vaccinations with intramuscular J8-DT/Alum. Vaccine-induced protection against an *S. pyogenes* skin challenge was assessed. J8-DT/HD-MAP vaccination led to a significant reduction in the number of *S. pyogenes* colony forming units in skin (92.9%) and blood (100%) compared to intramuscular vaccination with unadjuvanted J8-DT. The protection profile was comparable to that of intramuscular J8-DT/Alum. J8-DT/HD-MAP induced a shift in the antibody isotype profile, with a bias towards Th1-related isotypes, compared to J8-DT/Alum (Th2 bias). Based on the results of this study, the use of J8-DT/HD-MAP should be considered in future clinical development and control programs against *S. pyogenes*. Furthermore, the innate characteristics of the technology, such as vaccine stability and increased coverage, ease of use, reduction of sharp waste and the potential reduction of dose may be advantageous compared to current vaccination methods.

## Introduction

*Streptococcus pyogenes* (*S. pyogenes*/ group A *Streptococcus*/ GAS), is a Gram-positive, beta-haemolytic bacterial pathogen^1^ that causes a broad spectrum of human diseases. Infection causes significant global disease with over 18 million cases of severe disease^1^ and 500,000 deaths annually^2^. The most common infection sites are the skin and upper respiratory tract, resulting in impetigo and pharyngitis respectively^1^. Some of the highest rates of infection occur in Indigenous Australians^3,4^ (at least five times higher than non-Indigenous Australians)^3^, mostly associated with poverty, overcrowding and difficulties in accessing healthcare services^5^. Primary GAS infections can trigger post-infectious immune disorders^6^ such as acute rheumatic fever (ARF) and rheumatic heart disease (RHD), due to molecular mimicry of the *S. pyogenes* M-protein and human cardiac myosin^1,2,7^. Indigenous Australians are 20-times more at risk of succumbing to RHD than non-Indigenous Australians^8^. With emerging antimicrobial resistance becoming an increasing issue^9,10^, the need for a vaccine has never been more essential.

*S. pyogenes* has an array of virulence factors that contribute to its success as a pathogen. Vaccine candidates are aimed at disrupting and inhibiting these factors. Some of the potential candidates are based on C5a peptidase, streptococcal carbohydrate, fibronectin binding proteins, cysteine proteases, pyrogenic exotoxins and pili^11,12^. However promising, none have yet progressed to phase II clinical trials^13,14^. Other candidate vaccines target the M-protein (encoded by the *emm* gene), the major surface protein, which is anchored to the cell wall peptidoglycan and inhibits phagocytosis and promotes adherence to host epithelial cells^15^. Research into vaccine candidates based on peptides derived from the N-terminal domain of the M-protein was hampered due to safety concerns following a trial in 1969^16^, causing a ban for vaccine trials. Since lifting the embargo in 2006^17^, N-terminal M-protein vaccine candidates have made considerable progress. These candidates include fused recombinant peptides from the hypervariable N-terminal regions of M-proteins from multiple *S. pyogenes* strains^18–20^. The most recent, a 30 valent vaccine comprising four recombinant proteins, containing N-terminal peptides from 30 M proteins adjuvanted with alum, has completed phase I clinical trials^20^. *S. pyogenes* has a large diversity in *emm* types (over 240)^21,22^ and this may present a barrier to multivalent vaccine candidates, particularly in developing countries where even more extensive strain diversity is common^12^.

Another vaccine development approach uses the M-protein conserved sequence^12,23^. J8i is a minimal B-cell epitope comprised of 12 amino acids derived from the highly conserved C3-repeat domain of the M-protein^24^. When flanked by non-streptococcal helix promoting sequences to retain its native coiled-coil structure (which is required for immunogenicity and protective immune responses) the resulting 28-mer chimeric peptide is referred to as J8^24^. Genomic analysis of a number of Strep A isolates from Canada and within our laboratory collection has revealed that despite great allelic variation^25^ up to 94% of *S. pyogenes* isolates contain either J8 or the closely related allelic sequence, J8.1 in their *emm* gene^26–28^. Immunological cross-reactivity between the two allelic variants has been shown^26^. Further, J8 protects mice against intraperitoneal and skin infection with organisms bearing either the J8 or J8.1 allele^26,29–31^. When conjugated to diphtheria toxoid (DT) to create J8-DT, it is capable of stimulating T-helper cells and is immunogenic in multiple strains of mice^32^. J8 and J8-DT, when formulated with various adjuvants, have been shown to induce immune responses in mice that protect against multiple *S. pyogenes* strains in the skin, mucosa and deep tissue^29,32,33^. Various routes of vaccine delivery have been used, including subcutaneous, intramuscular and intranasal^12,32,33^. Mice immunised subcutaneously with J8-DT formulated with Alum (aluminium hydroxide) are protected against intraperitoneal^29,32^, intravenous^34^ and skin challenge^30,31^, which is mediated by J8-specific systemic antibodies. J8-DT delivered subcutaneously induces protection mediated by systemic antibodies^29^. However, cutaneous vaccination with J8-DT has not yet been investigated.

Skin-based immunisation routes have gained attention due to targeting of the epidermis and dermis layers rich in immune cells^35,36^. Several advantages are associated with cutaneous routes, particularly when using microarray patches (MAPs and HD-MAPs). These include dose sparing^37–42^, enhanced thermostability^41,42^, ease of use by healthcare workers and possibly recipients (if self-administered)^43^, reduced generation of sharp waste and risk of needle-stick injuries, good tolerability and enhanced acceptability in patients^44,45^. Cutaneous *S. pyogenes* vaccination with HD-MAPs may not only benefit vaccine efficacy (as HD-MAP targets the skin directly) but may also enhance vaccine coverage in areas/communities where disease is highly prevalent^2,3^.

HD-MAPs, developed by Vaxxas Pty Ltd, are at an advanced stage of development and have shown promising clinical trials results^42,44,45^. Compared to other MAP technologies used for clinical trials that use dissolving microprojections^43,46^, the Vaxxas HD-MAP is made of polymer as a solid array, making it suitable for large scale manufacturing. The thousands of projections per cm^2^ allow the targeting and drying of the vaccine at the tip of the microprojections, minimising the waste of vaccine. In clinical trials with Influenza, vaccine thermostability has been demonstrated when using HD-MAPs^42^.

The aims of this study were to compare vaccination with J8-DT via HD-MAP and adjuvanted intramuscular vaccination. Antibody responses, dose ranging, reduction in the number of vaccinations and protection from infection (skin challenge) were used to determine the performance of vaccination and establish the comparisons between the vaccination methods. The findings of this study should be considered when clinical trial testing and *S. pyogenes* control programs are being developed.

## Results

### J8-DT can be uniformly coated onto HD-MAPs with direct-jet printing, and the conjugate remains stable

**Fig. 1:**
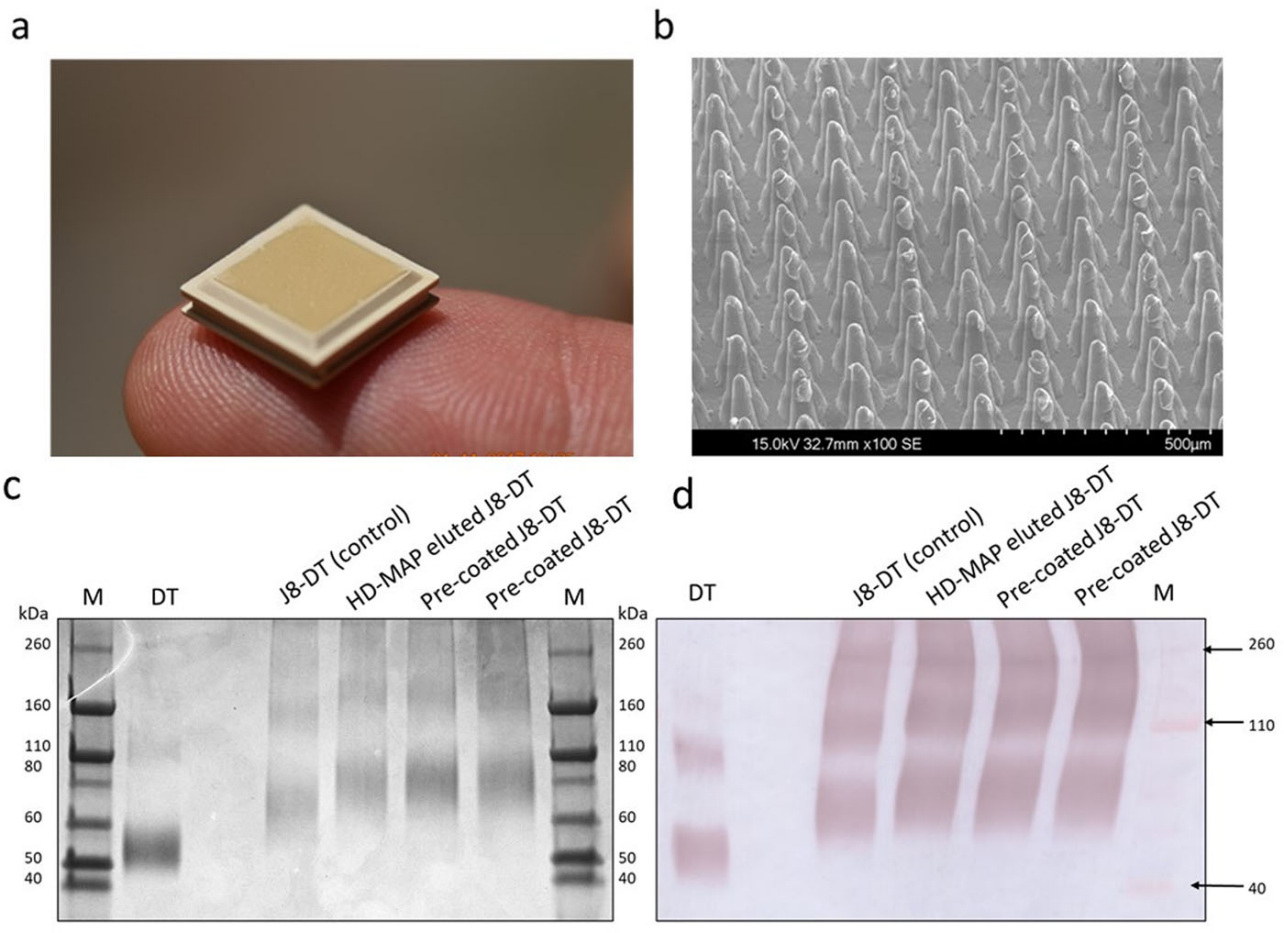
Coating and stability of HD-MAP coated vaccine. **a** Blank HD-MAPs with no coating were used as negative controls for immunogenicity. **b** J8-DT coating on HD-MAP microprojections via SEM analysis. **c** SDS-PAGE assessment of J8-DT eluted from HD-MAPs. SDS-PAGE (4–15% gel) demonstrating integrity of J8-DT conjugates 7 days post direct-jet printing onto HD-MAPs. HD-MAPs were coated with a theoretical load of 25 μg of J8-DT by direct-jet printing and eluted by agitation. The lanes contain unconjugated DT, control J8-DT, HD-MAP eluted J8-DT and J8-DT pre-coating onto HD-MAPs respectively. **d** Western blot analysis demonstrating binding of anti-J8-DT sera to DT or to J8-DT before and after coating and elution from HD-MAPs.

Blank HD-MAPs (no coating) were used for each immunogenicity study (Fig. 1a). HD-MAPs coated by direct-jet show the deposition of the vaccine drops at the tip of the projections on the scanning electron microscope (SEM; Fig. 1b). The material was coated onto the HD-MAPs without the addition of excipients. The coated material formed a uniform oval-shaped drop at the tip of each individual projection. Coated HD-MAPs were evaluated to determine the extent of vaccine loading with J8-DT and the stability of the conjugate on the HD-MAPs. MicroBCA analysis of eluted HD-MAPs showed that the coating process deposited a median loading per MAP for all vaccinations of 26.9 µg J8-DT (7% higher than the 25 µg theoretical load). SDS-PAGE was used to confirm the integrity of the peptide conjugate at the target time point of seven days post direct-jet printing (Fig. 1c, Supplementary Fig. 1a). This time point was considered as the maximum interval of time between HD-MAP coating and vaccination of the animals in this study and no longer time-points were tested as that was not an aim of the study. The SDS-PAGE (Sodium Dodecyl Sulphate Polyacrylamide Gel Electrophoresis) results demonstrated that the conjugate remained intact over that period as the bands of HD-MAP-eluted J8-DT were comparable to the uncoated J8-DT control. Therefore, no attempts to stabilise or add excipients to the vaccine antigen were performed as it remained stable through the printing and elution processes without the addition of other components.

Western blot with anti-J8-DT sera was used to confirm identity of HD-MAP eluted conjugates (Fig. 1d, Supplementary Fig. 1b). The binding of J8-DT anti-sera were comparable between control J8-DT, pre-coated J8-DT and HD-MAP eluted conjugate.

### J8-DT is immunogenic in vivo following HD-MAP coating and elution

**Fig. 2:**
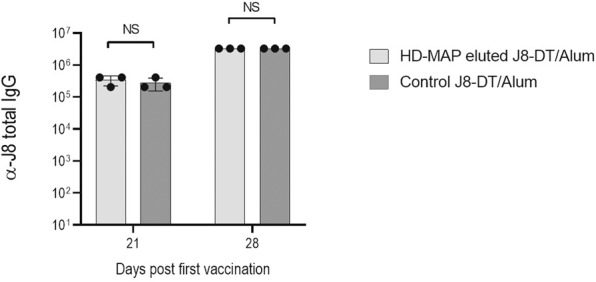
*α-*J8 total serum IgG titres using HD-MAP eluted antigen/Alum versus standard J8-DT/Alum. Groups of mice (*n* = 3) were vaccinated IM at days 0 and 21 with 30 µg of either standard J8-DT/Alum or HD-MAP-eluted J8-DT/Alum. Serum samples were collected at 21 and 28 days following the first vaccination. ELISA was used to determine α-J8 total IgG titres. Titres were defined as the highest dilution of serum for which OD was >3 standard deviations above the mean OD of control samples (PBS-vaccinated mice). *p* > 0.05 NS (no significance). Bars represent the mean ± SD.

Following confirmation that the peptide conjugate was biochemically stable on the HD-MAP over an 8-day period, the immune-stability of eluted material was assessed, by eluting the vaccine, immunising mice and measuring specific α-J8 total IgG antibodies. Similar IgG titres were elicited in the animals vaccinated with 30 µg HD-MAP-eluted J8-DT/Alum and 30 µg J8-DT/Alum standard vaccination groups, following the first and second vaccination (Mann-Whitney test, *p* > 0.05, Fig. 2). This confirmed the J8-DT conjugate retained its potency following the direct-jet coating.

### Local skin response in mice following HD-MAP application

**Fig. 3:**
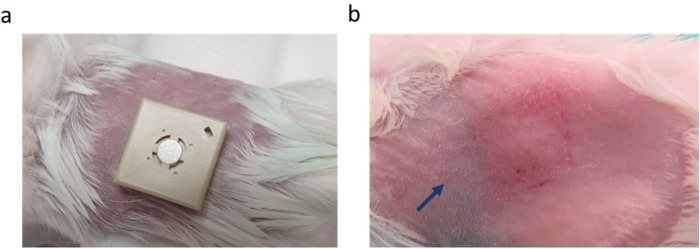
Typical erythema response post J8-DT/HD-MAP application. **a** HD-MAP application onto mouse flank. Prior to HD-MAP application animals were anaesthetised with an intraperitoneal injection of ketamine-xylazine (100 mg/kg and 20 mg/kg, respectively), depilatory cream (Nair™) was used to remove the hair from the animals, and HD-MAPs were applied with a spring-loaded applicator for 2 min and then removed manually. **b** Immediate erythema response following J8-DT coated HD-MAP application on the flank. Both uncoated and J8-DT coated HD-MAPs showed a similar erythema response, the image is representative of both coated and blank HD-MAP application. Photos were taken approximately one minute post HD-MAP removal from the skin. The arrow indicates the HD-MAP application site showing a mild erythema response.

Mice were then vaccinated with J8-DT-coated HD-MAPs. The applied HD-MAP covered most of the abdominal area of the flank of the mouse (Fig. 3a). The erythema response was mild in all animals after they received either a blank HD-MAP or the J8-DT-coated HD-MAP (Fig. 3b). Erythema at the application site was circumscribed to the skin surface under the HD-MAP (Fig. 3b). The application site marks were fully resolved by the time the next vaccination was due. In some cases, hair growth was increased only in the patched skin and by the time of the next vaccination hair had fully regrown at the application site.

### Antigen dose sparing of HD-MAP and IM delivered J8-DT evokes an antibody response

We determined if dose sparing or delivering a reduced number of HD-MAP vaccinations would induce antibody responses. The statistical analysis was tailored to address the effect of application method, antigen dose and number of vaccinations (HD-MAP application only) on the α-J8 IgG antibody titres.

**Fig. 4:**
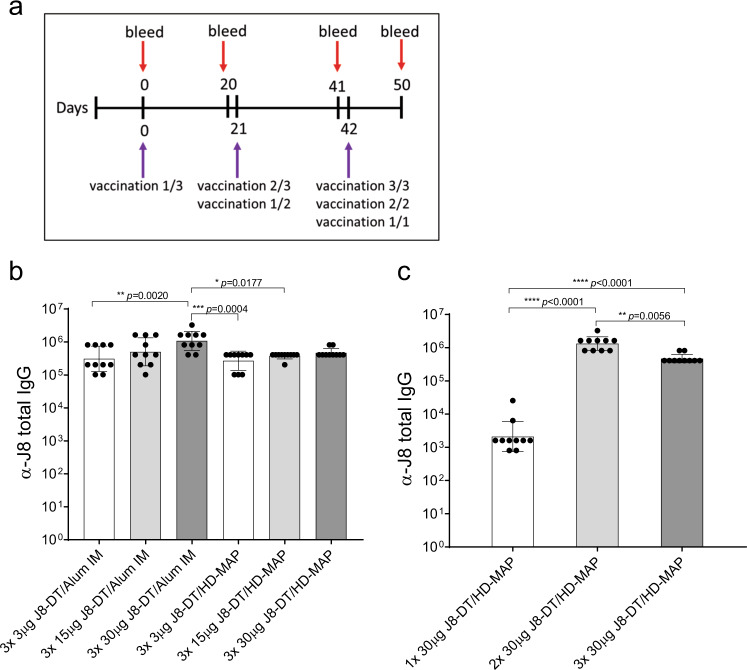
Immunogenicity of J8-DT given IM or via HD-MAPs at different doses and vaccination regimens. **a** Vaccination and sampling schedule. Groups of mice (*n* = 10) were vaccinated on day 0, 21 and 42 with either 3 µg, 15 µg or 30 µg J8-DT formulated with Alum (IM) or coated onto HD-MAPs as per the vaccination and sampling schedule. The total IgG α-J8 titres following three vaccinations with different vaccine doses (**b**) or following different number of vaccinations using the same dose (**c**), measured by indirect ELISA are shown. Bars represent geometric mean ± SD. Significance was determined using one-way ANOVA.

To determine the effect of the vaccination method and dose, the dose-matched groups for J8-DT/HD-MAP and J8-DT/alum IM were compared. There was a significant increase in total IgG when vaccinating with a higher dose (30 µg) for all time points (day 20, *p* = 0.0033; day 41, *p* < 0.0001; and day 50, *p* = 0.0005, two-way ANOVA, Supplementary table 1). The 30 µg J8-DT/Alum group induced significantly higher titres than 3 µg J8-DT/Alum, 3 µg J8-DT/HD-MAP and 15 µg J8-DT/HD-MAP IM at day 50 (Fig. 4b, *p* = 0.0020, *p* = 0.0004 and *p* = 0.0177, respectively, two-way ANOVA).

To test the effect on total IgG of the number of vaccinations, the groups vaccinated with one, two or three vaccinations of 30 µg J8-DT via HD-MAP were compared against each other. Remarkably, two 30 µg J8-DT/HD-MAP vaccinations induced higher total IgG titres at day 50 than one 30 µg J8-DT vaccination (*p* < 0.0001, one-way ANOVA) or three 30 µg J8-DT/HD-MAP vaccinations (*p* = 0.0056, one-way ANOVA) with (Fig. 4c). The three-vaccination group with 30 µg had higher titres than the one vaccination counterpart (*p* < 0.0001, one-way ANOVA).

**Fig. 5:**
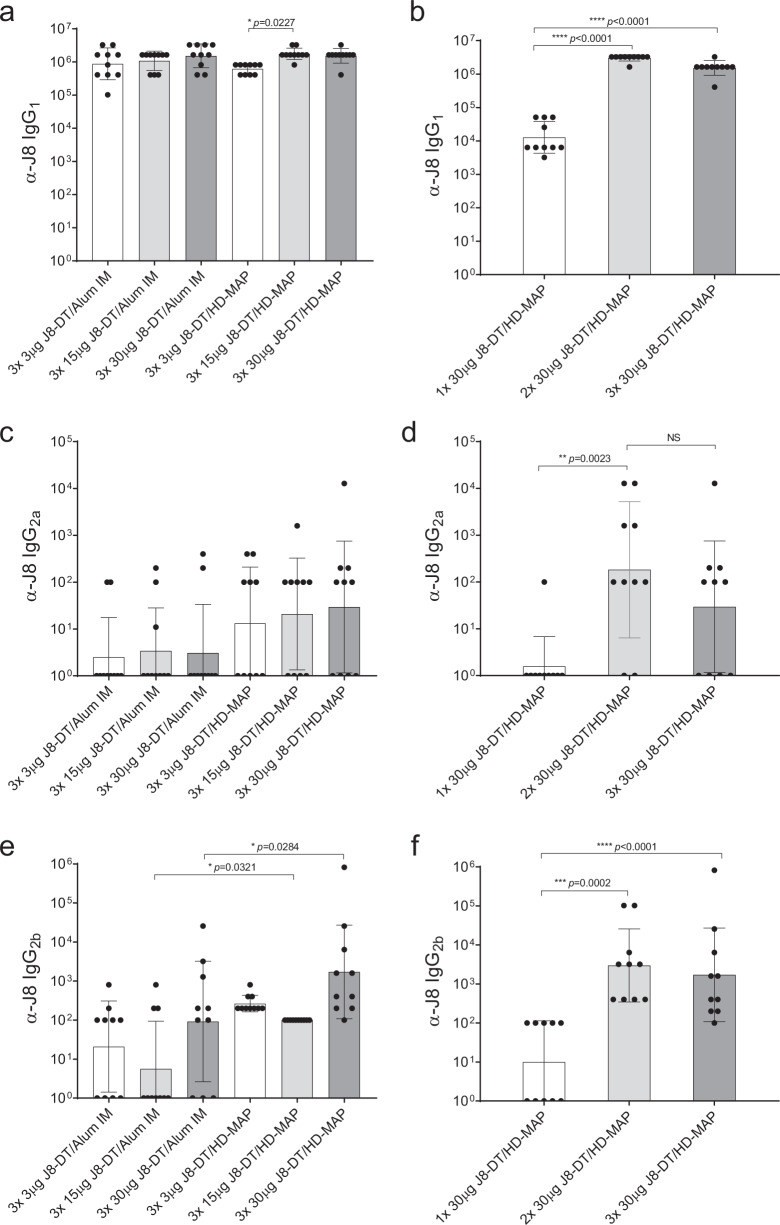
IgG isotype immunogenicity of J8-DT given IM or via HD-MAPs at different doses and vaccination regimens. Serum for IgG isotyping was performed on the samples taken at 8 days following final vaccination for all groups (Day 50). IM and HD-MAP dose-sparing groups were compared to determine the effect of dose and vaccination method using two-way ANOVA. Antibody isotyping was performed using indirect ELISA with isotype specific secondary IgG; α-J8 IgG isotype titres for IgG_1_ (**a**, **b**), IgG_2a_ (**c**, **d**) and IgG_2b_ (**e**, **f**) are shown. Bars represent geometric mean ± SD.

Regarding the serum IgG_1_ isotype responses, increasing the dose in MAP groups significantly raised the antibody response (*p* = 0.0032, two-way ANOVA). When comparing the HD-MAP and IM groups, the 15 µg J8-DT/HD-MAP group had significantly higher IgG_1_ titres than the 3 µg group (*p* = 0.0227, two-way ANOVA, Fig. 5a). For the assessment of the number of vaccinations with 30 µg J8-DT/HD-MAP, the one vaccination group had significantly lower IgG_1_ titres than the three (*p* < 0.0001, one-way ANOVA) and two vaccinations (*p* < 0.0001, one-way ANOVA) groups (Fig. 5b). Overall, there was an increased response with dose and number of vaccinations for the IgG_1_ response of the MAP groups.

For the IgG_2a_ response, there was an increase in the antibody response with HD-MAP compared to IM (*p* = 0.0057, two-way ANOVA). No differences were found between the matched dose IM vs HD-MAP groups (two-way ANOVA, Fig. 5c). The group that received two vaccinations had significantly higher titres than the group vaccinated once (*p* = 0.0043, one-way ANOVA, Fig. 5d).

IgG_2b_ responses were consistent in the HD-MAPs vaccinated groups with all animals eliciting specific α-J8 titres, whereas in the IM groups only 50% (15/30) of the animals had measurable IgG_2b_ titres. Although no significant differences were observed in the IM groups when comparing the different doses, there was an overall significant increase in the IgG_2b_ titres when increasing the antigen dose, independent of the application method (*p* = 0.0004, two-way ANOVA) and by applying the vaccine via HD-MAP (*p* < 0.0001, two-way ANOVA, Supplementary table 1). The HD-MAP groups were significantly higher at 15 µg (*p* = 0.0321, two-way ANOVA) and 30 µg (*p* = 0.0284, two-way ANOVA) compared to the IM counterparts (Fig. 5e). Regarding the number of vaccinations with 30 µg J8-DT/HD-MAP, one vaccination elicited significantly lower titres than two (*p* < 0.0001, one-way ANOVA) or three vaccinations (Fig. 5f, *p* = 0.0002, one-way ANOVA).

### J8-DT delivered by HD-MAP is immunogenic and protective against *S. pyogenes* skin and systemic infection

**Fig. 6:**
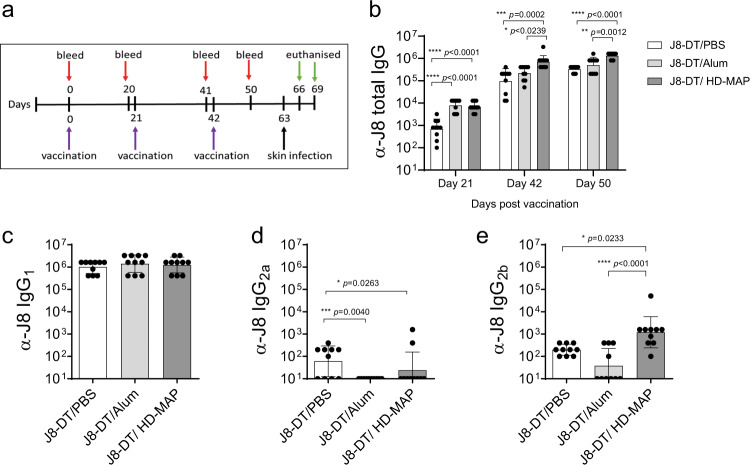
α-J8 specific IgG titres following vaccination with J8-DT/Alum, J8-DT/PBS, or J8-DT/HD-MAP. **a** Vaccination and sampling schedule. Mice (n = 10/group) were vaccinated with IM J8-DT formulated with either Alum, PBS or applied via HD-MAP on days 0, 21 and 42. Serum samples were collected on days 0, 20, 41 and 50 as per vaccination and sampling schedule. **b** Total α-J8 IgG measured at all blood sampling points. **c**–**e** IgG isotyping was performed using indirect ELISA with isotype specific secondary IgG. Isotyping for IgG_1_ **c**, IgG_2a_ **d**, and IgG_2b_ **e** was performed at the day 50 time point. Titres were defined as the highest dilution of serum for which the OD was > 3 standard deviations above the mean OD of the control samples (PBS-vaccinated mice). Bars represent the geometric mean ± SD. Significance was determined using one-way ANOVA.

We also compared J8-DT immunogenicity and protection when delivered via IM or HD-MAP at the 30 µg of antigen delivered three times. Although the three-times HD-MAP dose was slightly less immunogenic than the two-times HD-MAP dose, we chose the three-dose schedule for comparison because three doses of J8-DT/Alum are required for protection^47^. Total anti-J8 IgG titres were measured following each vaccination. After vaccination one and two, both the J8-DT/Alum IM and J8-DT/HD-MAP vaccinated groups (*n* = 10) elicited comparable total IgG anti-J8 IgG titres. Both groups (J8-DT/Alum and J8-DT/HD-MAP) also had significantly higher titres than J8-DT/PBS after the first vaccination (*p* < 0.0001 and *p* < 0.0001, respectively, one-way ANOVA). After the second and third vaccinations the J8-DT/HD-MAP had significantly higher α-J8 titres than both J8-DT/Alum (*p* = 0.0273 and *p* = 0.0012, respectively, one-way ANOVA) and J8-DT/PBS (*p* = 0.0002 and *p* < 0.0001, respectively, one-way ANOVA, Fig. 6b).

Serum IgG isotyping was undertaken with serum collected 8-days post final vaccination. IgG_1_ titres were comparable between all groups (mean antibody titre = ~10^6^, Fig. 6c). No IgG_2a_ was detected in the J8-DT/Alum group, with low but, significantly higher titres in the J8-DT/PBS group compared to the J8-DT/Alum group (*p* = 0.0004, one-way ANOVA) and J8-DT/MAP (*p* = 0.0263, one-way ANOVA) vaccinated groups (Fig. 6d). The J8-DT/HD-MAP group had significantly higher IgG_2b_ titres than both J8-DT/Alum and J8-DT/PBS (*p* < 0.0001, *p* = 0.0233, respectively, one-way ANOVA) IM groups (Fig. 6e). These immunogenicity results confirmed the data from the experiment presented in Figs 4, 5.

**Fig. 7:**
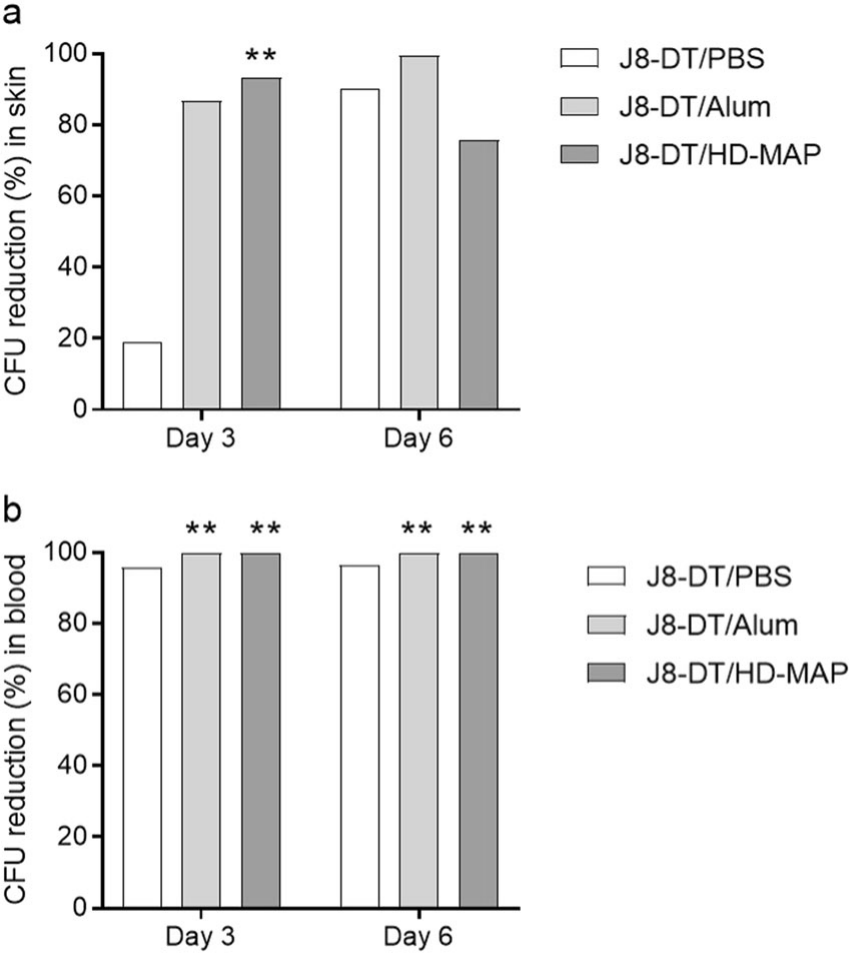
Protective efficacy of J8-DT HD-MAP against of *S. pyogenes* infection. Following 3 vaccinations with J8-DT/Alum, J8-DT/PBS or J8-DT/HD-MAP, groups of mice (*n* = 10) were infected via skin scarification with 1 × 10^6^ CFU/mouse. Mice were sacrificed on days 3 and 6 post-infection. **a** CFU percent reduction in skin samples. **b** CFU percent reduction in blood samples. Percent reduction was calculated by comparing the geometric mean of vaccinated groups CFU to the geometric mean CFU of the PBS/Alum group for the IM vaccinated groups (J8-DT/PBS and J8-DT/Alum); or the geometric mean of the blank HD-MAP group for the J8-DT/HD-MAP group. Graphic shows the CFU percent reduction in geometric mean. Individual CFU counts were used for the Mann-Whitney test to compare vaccinated groups with their respective controls. **p < 0.01.

Skin infection was induced using the *S. pyogenes* isolate pNS1 (*emm*100) to assess protection in the vaccinated animals. Animals were sacrificed day 3 and 6 post-infection. To assess protection, percent reduction in skin and blood bacterial burden geometric mean (expressed as colony forming units, CFU) comparison to the geometric mean CFU count of the control groups was determined (Fig. 7).

The geometric mean CFU in the skin samples for the control groups at day 3 and day 6 were for the PBS/Alum: 7.54 × 10^6^ and 2.14 × 10^4^, respectively; and 5.18 × 10^6^ and 3.34 × 10^3^ for the blank MAP group, respectively. On day 3 the J8-DT/HD-MAP group had a significant 93.27% reduction in skin bacterial burden (*p* = 0.0079, Mann–Whitney) compared to the blank MAP group (Fig. 7a). At day 6, CFU reduction was similar in all groups, with the J8-DT/PBS, J8-DT/Alum and J8-DT/HD-MAP having 90.35%, 99.67% and 75.87% CFU reduction in skin, respectively (Fig. 7a).

The geometric mean CFU counts in blood for the control groups at day 3 and day 6 were 1.50 × 10^4^ and 4.50 ×10^3^ for the PBS/Alum group, and 7.52 ×10^4^ and 1.18 ×10^4^ for the blank MAP group. No CFUs were detected in the blood samples from mice vaccinated with J8-DT/Alum or J8-DT/HD-MAP neither at day 3 nor day 6 (i.e. 100% CFU reduction compared to the control groups) (Fig. 7b).

## Discussion

A vaccine delivered through a mechanism, which is effective and minimally invasive is highly warranted for infections such as *S. pyogenes* that affect the young (school-age children) and the elderly. In addition, for a vaccine that is primarily aimed for developing countries or for low socio-economic settings, long shelf life is critical to its wide-spread distribution and usage. A vaccine delivered with HD-MAPs has the potential to offer such advantages as has been demonstrated previously^42^. J8-DT is one of the lead vaccine candidates for *S. pyogenes* and its formulation with Alum has been shown to induce specific antibody responses and protection against *S. pyogenes* challenges in a number of preclinical studies^29,30,32,34,47^. Additionally, the vaccine has also been successfully tested in a pilot human study^14^. The current study investigated its immunogenicity and efficacy with a view to developing an efficacious and efficient method of vaccination against *S. pyogenes* in humans and its subsequent use in vaccination control programs.

Drying and storage was a key aspect to determining conjugate integrity and whether J8-DT was suitable for use with HD-MAP vaccination. The initial immunogenicity study assessment with J8-DT, eluted off the HD-MAP followed by formulation with Alum, resulted in titres that were comparable to those induced by control J8-DT/Alum IM injection. This confirmed the integrity of the antigen’s epitopes after direct-jet printing and elution and their ability to mount a specific immune response. When comparing this to clinical studies, HD-MAPs coated with influenza vaccine using the direct-jet technology was shown to keep the antigen stable at different temperature conditions ranging from 2 to 8 °C to 40 ± 2°C for up to 12 months^42^. Moving forward with *S. pyogenes* antigens, studies purposely designed to test the stability of the antigen will be required to test HD-MAPs coated with J8-DT. Having a stable vaccine for long periods of time at temperatures higher than refrigeration will benefit vaccination against *S. pyogenes* in rural/isolated communities.

Not all the coated vaccine on the HD-MAP was transferred to the skin with the current iteration of the technology and further development is ongoing to reduce the proportion of unused vaccine. Vaccine delivery to the skin is affected by the target species and their innate anatomical and physiological characteristics. The HD-MAP technology is tailored for skin application to humans and to a depth of around 100–150 µm, and therefore, using it in small rodents may not represent its performance accurately in humans. Differences in the surface of the skin, skin layers, hair density and preparation of the skin prior to application may reduce the vaccine delivery in mice and may show a reduced performance of the device compared to the performance in humans. As the main purpose of these studies was to determine the immunogenicity of J8-DT using the HD-MAP technology, further assessment of vaccine transfer efficiency was not performed.

In this study, local skin responses following HD-MAP application showed mild erythema with no adverse effects to the mice following patch application and with full resolution of the application site before the next vaccination time point (<21 days). In humans, HD-MAP application without vaccine was shown to induce mild erythema^45^ with an increased reactogenicity when adding a vaccine to the HD-MAP^44^. HD-MAP application induces localised inflammation at the site of microneedle penetration^48^. This may be an advantageous response to vaccination as it triggers inflammatory responses in the host that may enhance immune responses. This controlled skin injury may be able to serve an adjuvant-like effect for HD-MAP vaccine delivery^48,49^. Skin has a high density of antigen presenting cells (APCs) and therefore serves as an efficient recipient tissue of vaccination^49^. With skin being one of the main routes of *S. pyogenes* infection, vaccination into this first-barrier tissue may ultimately enhance the host response to improve protection. Several studies have described the enhanced immunogenic response in mice vaccinated with HD-MAPs^37,41,50^.

Previous work with HD-MAP and other vaccines have reported dose sparing with increased immunogenicity in preclinical models^37,38,40,50^. Although, no dose sparing was seen either in the Alum-adjuvanted IM and HD-MAP groups, two vaccinations with J8-DT/HD-MAP could be explored further as animals in this group had either comparable or higher total IgG responses than the groups vaccinated with three-vaccination regimen with 30 µg J8-DT/Alum IM and J8-DT/HD-MAP. This could be an improvement to the vaccination scheme used in previous studies^29,32^, as the same titre of antibody response is achieved with one less vaccination in this HD-MAP group. However, this remains to be shown as experiments with J8-DT/Alum show that three doses are required for protective efficacy^47^. Current human data suggest that influenza vaccination with HD-MAP not only induces similar protective responses to those induced by needle and syringe vaccination but may also show a dose-sparing effect^42^. With the findings of this study and clinical trial data, HD-MAP vaccination may potentially reduce the total vaccine amount required to have a similar effect to needle and syringe vaccination. With that improvement, vaccine supply could increase the coverage in emerging markets, reduce the cost per vaccinated individual as well as lower storage and transportation costs (less vaccine required), increasing the efficacy of vaccination programs. Further studies may investigate this phenomenon to determine if indeed, a two-vaccination regimen can replace the current standard three vaccinations in the mouse model.

The direct comparison between J8-DT/HD-MAP, J8-DT/Alum IM and J8-DT/PBS at a matched dose resulted in a similar antibody response profile with significant improved protection at the earlier culling point (day 3) in the J8-DT/MAP group, following a three-vaccination regimen in mice. After a single vaccination, J8-DT/HD-MAP group had significantly higher total IgG titres than J8-DT/PBS, which suggest accelerated kinetics in establishing an immune response. In the case of HD-MAP vaccination, this feature is like what has been described for influenza vaccination in humans^42^. In that study, groups of subjects vaccinated with HD-MAPs at a matched dose or 2/3 of the dose had between 30–42% higher seroconversion rate than IM vaccinated subjects at day 8 post vaccination.

In the direct comparison with IM applications, J8-DT/HD-MAP group had a significantly higher total IgG response than both IM J8-DT/PBS and J8-DT/Alum following the last vaccination. This was consistent with CFU reduction in skin by day 3 post challenge, despite the relatively low sample size. Both the J8-DT/HD-MAP and J8-DT/Alum groups had 100% reduction in blood bacterial burden, indicating comparable efficiency of HD-MAP and IM vaccination against invasive infection. Taken together, the post challenge data from both skin and blood demonstrate that J8-DT/HD-MAP had similar efficacy to that of J8-DT/Alum IM without the addition of the Alum adjuvant. Not only did HD-MAP vaccination induce strong antibody responses but it also protected against live challenge with the target microorganism with an efficacy of the HD-MAP comparable, if not better than the IM administered vaccine.

The HD-MAP application was performed on the flank of the mouse due to availability of space and the preferred application onto a flat surface without compromising the device performance in relation to human applications. In clinical trials, vaccination with HD-MAPs showed similar responses in subjects vaccinated in the forearm and upper arm regions^42^. We therefore expect that different vaccination sites do not affect the generated antibody responses. When considering anatomical site for skin infection, the nape of the neck was chosen to reduce the likelihood that animals will directly contact the challenged skin area^30^. Furthermore, by challenging in a different area of skin to where the vaccine was applied shows that the protective response was not localised to the site of vaccine application.

Previous studies with J8-DT/Alum in mice reported similar total IgG titres (10^6^) following three IM vaccinations^32^, with IgG_1_ as the dominant isotype^34^. The findings of the current study reinforce these data for J8-DT/Alum and show that the J8-DT/HD-MAP induces similar total IgG responses without the addition of the adjuvant. Nevertheless, animals vaccinated with J8-DT/HD-MAP consistently had significantly higher IgG_2b_ titres than their J8-DT/Alum counterparts. Albeit not statistically significant, a higher proportion of the animals had a measurable specific IgG_2a_ response in the J8-DT/HD-MAP vaccinated groups compared to the matched IM counterparts. A bias towards IgG_2_ response is characteristic of a cellular Th1 response^51^, which will induce the secretion of IL-2, interferon-γ (IFN-γ) and lymphotoxin-α. IFN-γ is required to protect mice against lethal skin infections and can also stop the dissemination of bacteria to local tissue^52^, and may play a role in the CFU reduction observed in this study. It has been demonstrated that HD-MAP vaccination targets a cellular immune response^39,53^. Similarly, our data also demonstrate that unadjuvanted J8-DT delivered via HD-MAP targets local immune cells (APCs) in the cutaneous layers and induces Th1/Th2 responses. Interestingly, unadjuvanted J8-DT delivered via IM route induced significantly higher IgG2a (Th1) response in comparison to unadjuvanted J8-DT delivered on HD-MAP. We believe that this is due to the different route of antigen delivery and thus targeting of different immune/antigen presenting cells. Unadjuvanted peptide conjugate (J8-DT) delivered via IM route, due to their small size is likely to stimulate B cells residing in the follicles and thus induce a response which is not seen by HD-MAP delivered J8-DT. The targeting of B cells by small molecules (<70 kDa) has been previously reported^54^.

We can speculate on the biological significance of J8-DT/HD-MAP vaccination. In humans, it is described that *S. pyogenes* resistance to pharyngitis is generally higher in adults^55^ and differences in the IgG subtype profile between children and adults may explain the reason for this. Mortensen et al.^55^ demonstrated that adults express higher levels of IFN-γ and IgG_3_ (Th1-related), which may be responsible for less susceptibility to *S. pyogenes* infection in adults. Taking this into account, an optimal J8-DT vaccine should probably aim to achieve similar IgG profiles of those less-susceptible-to-infection human groups. In this study, J8-DT/HD-MAP vaccination induced antibodies that are characteristic of the Th1 type responses and as such could lead to cellular responses capable of targeting *S. pyogenes*. Moreover, Th1 immune responses have been shown to promote protection against systemic invasive *S. pyogenes* infection^56^.

The finding that groups vaccinated via HD-MAP induced similar total IgG titres to IM and a differentiated (and probably desirable) IgG subtype response, presents as an opportunity for *S. pyogenes* vaccination, as removing Alum from the formulation may reduce the vaccine manufacturing costs and indirectly improve the coverage of vaccination by making it less reactogenic and more accessible. It has been reported that Alum can induce minor side effects such as pruritic nodes at injection sites^57^ and granulomatous reactions in humans^58^. By removing it from the vaccine formulation, the risk of these occurrences will be reduced. Adding this to innate advantages of the HD-MAP technology (potential removal of cold chain, single use, ease of use by health workers, self-administration) may open new possibilities when designing control campaigns for *S. pyogenes* vaccination. Furthermore, reduced number of vaccinations, with performance comparable to a three-dose regimen would also reduce the cost of vaccination campaigns both from the total vaccine costs per individual, health worker involvement and the logistic costs that involve an extra vaccination. Patient compliance will most likely increase as it is easier to attend to a lower number of vaccinations.

In summary, this study shows that HD-MAP vaccination with J8-DT induces comparable humoral responses to adjuvanted intramuscular vaccination. In addition, both the vaccine approaches demonstrated comparable efficacy in protection against *S. pyogenes* skin and invasive infections. A reduced number of vaccine doses were shown to achieve comparable immune responses, although efficacy was only compared using the three-dose regimens. The results described herein provide a proof-of-principle for further investigation in vaccination against *S. pyogenes* in preclinical models and in clinical trials using the HD-MAP. Not only does the MAP technology have an immunological advantage as shown in this study, but it may also benefit the planning and execution of campaigns to control *S. pyogenes* diseases in areas where they are prevalent.

## Methods

### Ethical statement

Mice were housed at Griffith University’s Animal Facility (Gold Coast, Australia). All experiments and methods for animal procedures were approved by the Griffith University Animal Ethics Committee (Animal Ethics Approval GLY/17/16) in compliance with Australian National Health and Medical Research Council Guidelines. Mice were monitored daily for general health. Following challenge, mice were monitored for signs of illness as per a score sheet approved by Griffith University Animal Ethics Committee.

### Peptide synthesis and conjugation

The J8 peptide (QAEDKVKQSREAKKQVEKALKQLEDKVQ with a cysteine residue at the C-terminus) was synthesised by China Peptides (China) and conjugated to diphtheria toxoid as previously described^29,59^. Briefly, MCS (6-Maleimido-Caproyl n-Hydroxy Succinimide; Sigma, USA) was dissolved in DMF (dimethylformamide; Sigma, USA). DT was added with slow mixing for one hour and unbound MCS was removed with overnight dialysis. The J8 peptide was added to the activated carrier, followed by further dialysis. The protein concentration of the peptide conjugate was estimated using Bicinchoninic acid (BCA, Thermofisher USA), following manufacturer’s instructions. The molecular weight of DT is ~60 kDa and J8 ~3 kDa. To confirm conjugation, and to determine the peptide-protein ratio, DT and J8-DT samples are sent to the Australian Proteome Analysis Facility (APAF, NSW). The amino acid analysis reports the conjugation ratio of J8 to DT, and we routinely get a loading ratio of 1:8–12 (where one molecule of DT is conjugated to 8–12 J8 units)^60^. The conjugates were either stored lyophilised or in solution at −20 °C.

### HD-MAPs and coating using direct-jet process

HD-MAPs were manufactured by injection moulding of polymer, to produce HD-MAPs of 10 × 10 mm with approximately 3136 projections per patch. Each projection was approximately 250 µm high, 120 µm wide at the base and had a sharp point of < 25 µm. HD-MAPs follow the same specification as those used in a human clinical study with influenza vaccination^42^. The vaccine was applied to the tips of HD-MAPs using a “direct-jet” process (Vaxxas Pty Ltd, Australia) that deposits individual droplets onto the tip of each projection. HD-MAPs were coated with a theoretical dose of ~25 µg J8-DT, to deliver ~15 µg per HD-MAP at a 60% vaccine transfer efficiency; or, were left uncoated (blank HD-MAP with no vaccine) for control groups. Following coating, HD-MAPs were placed into 12-well plates, foil-sealed, and stored at 2–8 °C with desiccant until use. The J8-DT-coated HD-MAPs were used within one week of coating following experimental verification by testing J8-DT’s stability (Fig. 1).

### Determination of protein concentration and conjugate stability on MAPs

To confirm the loading of vaccine onto the HD-MAPs, they were eluted in Dulbecco’s Phosphate Buffered Saline (DPBS; Sigma-Aldrich) and analysed using the micro Bicinchoninic acid (microBCA; Thermofisher, USA) assay. Diluted standards of J8-DT were used to interpolate the J8-DT loading per HD-MAP and to provide an accurate quantitation. Absorbance measurements were taken on a Spectrostar Nano spectrometer (BMG Labtech, Germany) at 562 nm.

The stability of the J8-DT conjugate following direct-jet coating and drying onto the HD-MAPs was also assessed through SDS-PAGE. SDS-PAGE was performed using a pre-cast mini-PROTEAN 4–15% Bis–Tris polyacrylamide gel (Bio-Rad, USA) run under reducing conditions and stained with Pierce Silver Stain Kit (Thermo, USA). Pre-coated J8-DT conjugate and control J8-DT were included as a comparison to show stability of the conjugate following coating on the HD-MAP.

Western blot was used to assess the binding capabilities of J8-DT antibodies to HD-MAP eluted conjugate. The SDS-PAGE gel was transferred to a 0.45 µm nitrocellulose membrane (Bio-Rad, Germany) and incubated with anti-J8-DT sera. Blots and gels shown derive from the same experiment and were processed in parallel.

### Mouse vaccinations, MAP applications and blood sampling

BALB/c mice (female, 4-8 weeks old) were sourced from the Animal Resource Centre (ARC, WA, Australia). BALB/c mice were vaccinated intramuscularly (IM) in the hind leg or via HD-MAP on the flank. For IM vaccination, mice were physically restrained while receiving the vaccine formulation (50 µl) using a 26½” needle in the thigh muscle. Before injecting the vaccine, mild pressure was applied to the syringe plunger to verify vaccine was not being applied to a blood vessel. IM J8-DT vaccinations were formulated with aluminium hydroxide (Alum; 2% Alhydrogel, Brenntag Biosector, Denmark) or PBS in a 1:1 volume ratio.

Prior to HD-MAP vaccination, mice were anesthetised with an intraperitoneal (IP) injection of ketamine (100 mg/kg) and xylazine (20 mg/kg). The fur from the flank of the animal was removed with clippers and then commercial depilatory cream (Nair™ Sensitive), applied for one minute, before removal with a soft dampened cloth. Following removal of the cream, the skin surface was dried with paper tissue. With the animal in the lateral decubitus position and exposing the hairless flank skin, two HD-MAPs were applied using a spring-loaded applicator, one to each flank, to deliver a total dose of 30 µg (15 µg per HD-MAP); one HD-MAP to deliver a dose of either 15 µg or 3 µg (depending on the assigned group), or un-coated blank HD-MAPs. The HD-MAP was left on the skin for 2 min, to allow the vaccine to be absorbed by the epidermis and dermis skin layers, before being removed.

Blood was collected one day prior to each vaccination by tail bleed and seven days post final vaccination via the submandibular vein. The neat blood was left to clot at 4 °C for at least 4 h prior to serum collection for antibody determination by ELISA.

### Antibody response determination by indirect Enzyme-linked Immunosorbent assay (ELISA)

Indirect-ELISA was used to quantify antigen-specific anti-J8 IgG antibody titres as described elsewhere^61^. Goat anti-mouse IgG Horseradish peroxidase (HRP) linked antibodies (Bio-Rad, Australia) were used to detect antigen-specific antibodies. Optical density (OD) at 450 nm was measured with a Tecan Infinite m200 Pro plate reader. Titres were defined as the highest dilution of serum for which the OD was > 3 standard deviations (SD) above the mean OD of control samples (PBS and blank HD-MAP-vaccinated mice).

### Bacterial strains and culture media

*S. pyogenes* pNS1 (*emm*100) was obtained from the Menzies School of Health Research (Darwin, NT, Australia). The isolate had previously been serially passaged in mice to ensure virulence. To prepare for challenge, the isolate was grown overnight in a liquid Todd-Hewitt broth medium (Oxoid, Australia) supplemented with 1% yeast and 1% neopeptone (Difco, Australia). To determine the number of colony forming units (CFUs), the overnight culture was 10-fold serially diluted, plated in duplicate on Columbia Blood Agar (CBA; Oxoid, UK) and supplemented with 5% defibrinated horse blood (Equicell, Australia). The culture was then adjusted to obtain the optimal inoculum^30^ for skin challenge.

### Superficial skin challenge infection

Mice were infected using a skin scarification model as previously described^30^. Mice were anesthetised with an IP injection of 100 mg/kg and xylazine 20 mg/kg. The fur from the nape of the neck was removed using clippers and then superficially scarified. A 10 µl inoculum containing 1 × 10^6^ CFUs was topically applied. Once the inoculum had absorbed into the skin, a temporary cover (Band-Aid™) was applied to the wound and mice were housed individually. Mice were monitored daily for signs of illness as per a score sheet approved by Griffith University Institutional Biosafety Committee.

### Sample collection and CFU quantification

At days 3 and 6 post-infection, a defined number of mice (five mice per time point) from each group was sacrificed via CO_2_ inhalation. Whole blood was collected via cardiac puncture into tubes containing Ethylenediaminetetraacetic acid (EDTA). Skin from the infection site (2–3 mm surrounding the lesion) was manually collected using dissection equipment. Skin was mechanically homogenised using Bullet Blender™ (Next Advance) following the manufacturer’s instructions. Samples were serially diluted (10-fold to 10^3^ for blood and 10^6^ for skin) and plated in replicates onto streptococcal selective CBA plates. Following overnight incubation at 37 °C, CFU were counted to determine bacterial load.

### Initial immunogenicity assessment in BALB/c with J8-DT

An initial assessment of the stability of the vaccine material coated onto the HD-MAP and subsequent elution was undertaken in BALB/c mice. Following J8-DT coating, HD-MAPs were stored at 4 °C in a sealed foil pouch with desiccant for up to 7 days. Following the manual elution of vaccine conjugate material from the HD-MAP (HD-MAP-eluted), groups of three BALB/c mice received two 30 µg IM vaccinations (50 µl of vaccine solution) of either HD-MAP-eluted J8-DT plus Alum (added fresh) or standard J8-DT formulated 1:1 with Alum, respectively. Vaccinations were given on day 0 with either fresh J8-DT/Alum or freshly eluted J8-DT from HD-MAP/Alum. For the second vaccination at day 28, animals were vaccinated either with freshly made J8-DT/Alum; or, for the eluted HD-MAP group, with frozen and thawed material stored for approximately 28 days post-elution with added Alum. The serum collection was performed at days 20 and 28 after the first vaccination to assess antigen-specific serum IgG titres.

### J8-DT/Alum and J8-DT/HD-MAP dose sparing and J8-DT/HD-MAP vaccination regimen comparison (number of vaccinations)

Eighty female BALB/c mice (4–6 weeks old) were allocated into eight groups of 10 animals each. To assess dose-sparing concentrations of antigen, three groups of mice received three vaccinations with either 3 µg, 15 µg or 30 µg of J8-DT/Alum administered IM; three dose-matched groups received J8-DT/HD-MAP applications using the same vaccination scheme. For these groups, vaccinations were given at days 0, 21 and 42. To assess if the number of vaccinations with the same J8-DT dose (30 μg J8-DT) had an effect on the antibody responses, two groups received either one or two vaccinations with 30 µg J8-DT/HD-MAP. For these groups, vaccinations were given either at day 21 and 42, or on day 42 for the two and one vaccination groups, respectively. All mice were bled one day prior to each vaccination and 8 days post final vaccination.

### Immunogenicity and protection against skin *S. pyogenes* challenge comparing J8-DT/Alum and J8-DT/HD-MAP at optimal vaccination dose

Fifty female BALB/c mice (4–6 weeks old) were allocated into five groups of 10 animals each. The first test group received a total of 30 µg J8-DT applied over two HD-MAPs (J8-DT/HD-MAP, estimated loading per HD-MAP of 25 µg with a 60% vaccine transfer efficiency), the second test group was given 30 µg J8-DT in PBS (J8-DT/PBS) IM, and the third test group was given 30 µg J8-DT formulated with Alum (J8-DT/Alum) IM. As negative controls, one group received un-coated HD-MAPs and another group was given PBS/Alum IM. Vaccinations were given on days 0, 21 and 42. All animals received a skin challenge with *S. pyogenes*, 21 days following the last vaccination. Five animals per group were euthanised at days 3 and 6 post skin challenge. Blood samples were taken one day before each vaccination, and eight days post final vaccination.

### Statistical analysis

Descriptive statistics of results were made using GraphPad Prism version 7. Antibody titres were transformed into logarithmic of two (log_2_) scale for statistical comparisons as the indirect ELISA assay uses 2-fold serial dilutions. However, original antibody titres are plotted to simplify the graphical description. Statistical differences between the bacterial load of two groups were determined using either *t*-Student or Mann–Whitney tests. One-way ANOVA test was used to compare differences between three or more groups. Two-way ANOVA was used to compare the matched IM and HD-MAP dose-sparing groups at three different doses (3, 15 and 30 μg J8-DT). For multiple comparisons, the Bonferroni correction was used. A confidence level of 95% was used to determine statistical significance.

### Reporting summary

Further information on research design is available in the Nature Research Reporting Summary linked to this article.

## Supplementary information

Supplementary Information

Reporting Summary

## Data Availability

All data needed to draw the conclusions in the paper are present in the paper and/or the supplementary material. Additional data related to this paper may be requested from the authors.
