# Author Correction: M-protein based vaccine induces immunogenicity and protection from *Streptococcus pyogenes* when delivered on a high-density microarray patch (HD-MAP)

**DOI:** 10.1038/s41541-020-00233-z

**Published:** 2020-09-03

**Authors:** Jamie-Lee S. Mills, Cesar M. Jayashi Flores, Simone Reynolds, Christine Wun, Ainslie Calcutt, S. Ben Baker, Senthil Murugappan, Alexandra C. I. Depelsenaire, Jessica Dooley, Paul V. Fahey, Angus H. Forster, Manisha Pandey, Michael F. Good

**Affiliations:** 1 Institute for Glycomics, Griffith University, Gold Coast, Australia; 2 Vaxxas Pty Ltd, Translational Research Institute, Woolloongabba, Australia

Correction to: *npj Vaccines* 10.1038/s41541-020-00222-2, published online 7 August 2020

In the original version of this Article, an author’s name was misspelled. This has now been corrected in both the HTML and PDF versions of the Article.

