# Supplementary material for: M-protein based vaccine induces immunogenicity and protection from Streptococcus pyogenes when delivered on a high-density microarray patch (HD-MAP)
Source: NPJ Vaccines. 2020 Aug 7;5:74. doi: 10.1038/s41541-020-00222-2 (PMC7414110; doi:10.1038/s41541-020-00222-2)

Supplementary Information

Supplementary Table 1. Statistical analysis: J8-DT/Alum and J8-DT/MAP dose sparing and J8-DT/HD-MAP vaccination regime comparison (number of vaccinations)

**Two-way ANOVA**	**Day 21 (p value, significance) †**	**Day 41 (p value, significance) †**	**Day 50 (p value, significance) †**			
**Effect**	Total IgG	Total IgG	Total	IgG1	IgG2a	IgG2b
**Interaction**	0.0631 NS	0.4194 NS	0.2414 NS	0.0579 NS	0.9292	0.8097 NS
**Application method**	0.0290 *	0.1611 NS	0.0213 *	0.1956	0.0057 **	< 0.0001 ****
**Dose**	0.0033 **	< 0.0001 ****	0.0005 ***	< 0.0001 ****	0.8093 NS	0.0004 ***

† NS: non-significant, $p > 0.05$; * $p < 0.05$; ** $p < 0.01$; *** $p < 0.001$; **** $p < 0.0001$.

Post hoc Bonferroni's multiple comparisons test

**Comparison Total IgG**	**Day 21 (p value, significance) †**	**Day 41 (p value, significance) †**	**Day 50 (p value, significance) †**
3µg J8-DT/Alum IM vs 3µg J8-DT/MAP	0.0631 NS	> 0.9999 NS	> 0.9999 NS
15µg J8-DT/Alum IM vs 15µg J8-DT/MAP	0.0290 *	0.2113	> 0.9999 NS
30µg J8-DT/Alum IM vs 30µg J8-DT/MAP	0.0033 **	> 0.9999 NS	0.0250 *

† NS: non-significant, $p > 0.05$; * $p < 0.05$; ** $p < 0.01$; *** $p < 0.001$; **** $p < 0.0001$.

Supplementary Figure 1: Full image of SDS-PAGE and Western blot from figure 1 c and d: (a) SDS-PAGE assessment of J8-DT eluted from HD-MAPs. SDS-PAGE (4-15% gel) demonstrating integrity of J8-DT conjugates 7 days post direct-jet printing onto HD-MAPs. HD-MAPs were coated with a theoretical load of 25 μg of J8-DT by direct-jet printing and eluted by agitation. The lanes contain unconjugated DT, control J8-DT, HD-MAP eluted J8-DT and J8-DT pre-coating onto HD-MAPs respectively. **(b)** Western blot analysis demonstrating binding of anti-J8-DT sera to DT or to J8-DT before and after coating and elution from HD-MAPs.

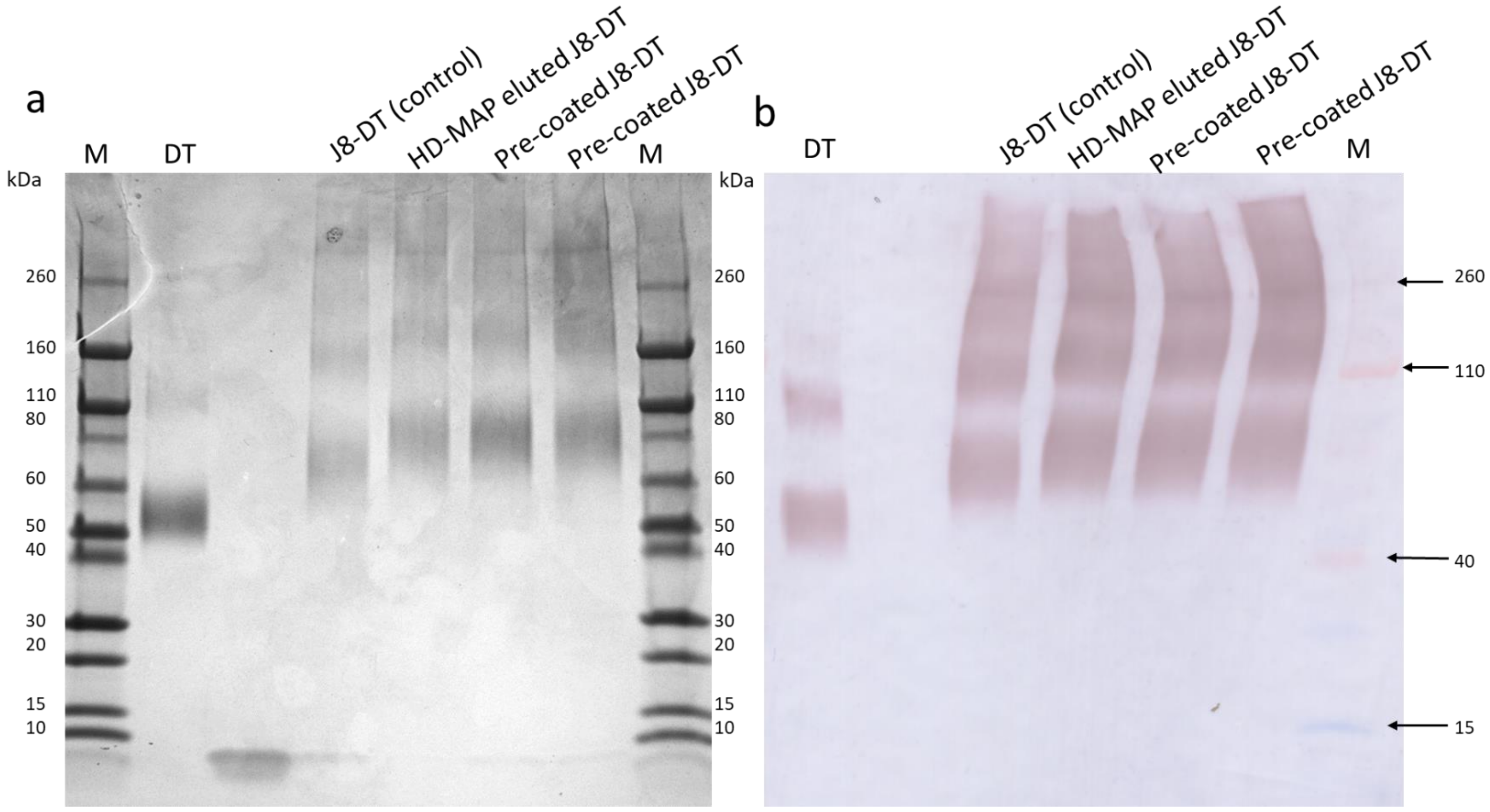

Supplement: Supplementary file 1 — Supplementary Information [file 41541_2020_222_MOESM1_ESM.pdf]
